# A Bayesian spatio‐temporal approach for real‐time detection of disease outbreaks: a case study

**DOI:** 10.1186/s12911-014-0108-4

**Published:** 2014-12-05

**Authors:** Jian Zou, Alan F Karr, Gauri Datta, James Lynch, Shaun Grannis

**Affiliations:** Worcester Polytechnic Institute, Worcester, USA; RTI International, Research Triangle Park, USA; University of Georgia, Athens, USA; University of South Carolina, Columbia, USA; Indiana University, Indianapolis, USA

**Keywords:** Conditional autoregressive process, Influenza, Gaussian Markov random field, Spatial statistics, Spatio‐temporal, Syndromic surveillance

## Abstract

**Background:**

For researchers and public health agencies, the complexity of high‐dimensional spatio‐temporal data in surveillance for large reporting networks presents numerous challenges, which include low signal‐to‐noise ratios, spatial and temporal dependencies, and the need to characterize uncertainties. Central to the problem in the context of disease outbreaks is a decision structure that requires trading off false positives for delayed detections.

**Methods:**

In this paper we apply a previously developed Bayesian hierarchical model to a data set from the Indiana Public Health Emergency Surveillance System (PHESS) containing three years of emergency department visits for influenza‐like illness and respiratory illness. Among issues requiring attention were selection of the underlying network (Too few nodes attenuate important structure, while too many nodes impose barriers to both modeling and computation.); ensuring that confidentiality protections in the data do not impede important modeling day of week effects; and evaluating the performance of the model.

**Results:**

Our results show that the model captures salient spatio‐temporal dynamics that are present in public health surveillance data sets, and that it appears to detect both “annual” and “atypical” outbreaks in a timely, accurate manner. We present maps that help make model output accessible and comprehensible to public health authorities. We use an illustrative family of decision rules to show how output from the model can be used to inform false positive–delayed detection tradeoffs.

**Conclusions:**

The advantages of our methodology for addressing the complicated issues of real world surveillance data applications are three‐fold. We can easily incorporate additional covariate information and spatio‐temporal dynamics in the data. Second, we furnish a unified framework to provide uncertainties associated with each parameter. Third, we are able to handle multiplicity issues by using a Bayesian approach. The urgent need to quickly and effectively monitor the health of the public makes our methodology a potentially plausible and useful surveillance approach for health professionals.

## Background

Syndromic surveillance uses syndrome (a specific collection of clinical symptoms) data as indicators of a disease outbreak, and monitors syndromes in public health‐related information sources for early detection of adverse disease events. Many health agencies are adopting and implementing syndromic surveillance systems. These systems meet a critical need for effective prevention, detection and management of infectious disease outbreaks, which occur either naturally or by bioterrorism attacks. However, there are numerous challenges in developing such systems, including: (i) incorporating situation‐specific characteristics such as covariate information for certain diseases; (ii) accommodating the spatial and temporal dynamics of the disease; (iii) integrating data from multiple sources; and (iv) providing analysis and visualization tools to help detect unexpected patterns. New methods that improve the overall detection capabilities of these systems while also minimizing the number of false positives can have a broad social impact.

There exists a plethora of surveillance methods in the literature. One of the methods widely used by public health departments is the CUSUM chart [1]. It was developed specifically to detect changes in patterns over time. Other variants followed in the areas of quality control and disease surveillance [2‐5]. These are constructed by cumulative recording of events over time. The CUSUM technique detects shifts in single or multiple parameters while usually assuming the target parameters are constant. However, disease incidences, as well as their associated background counts, vary naturally in space and time. Techniques that do not account for these spatial and temporal dynamics, such as the CUSUM, can lead to unsatisfactory results for syndromic surveillance purposes.

Spatial heterogeneities occur naturally when the study involves a large geographical area. For instance, strong correlations emerge between the infectious individuals and their interactions, which are usually spatially aggregated. One can capture the wave‐like spread of invading diseases within a population by using certain time series models. There are also heterogeneities between distinct populations, such as different towns and cities, or different geographic regions. Models for such scenarios must incorporate the correlation between the populations and the effects of the transmission between them. To accommodate this, a network of sites/nodes is assumed where dependencies among adjacent sites are modelled with spatial correlations and edges between sites determine the adjacency structure of the network. In our examples, sites are a collection of counties (regional labor markets) and “adjacent” means geographically contiguous or “sharing a common border”. Data are syndrome counts attributed to the nodes in the network throughtime.

In the surveillance context, spatial scan statistics [6] have been applied to a wide variety of epidemiological studies for disease cluster detection. However, this method lacks measures of uncertainty associated with the identified clusters, and it is unable to account for covariate information. Bayesian hierarchical models have become increasingly popular in the analysis of spatial and spatio‐temporal data. Banks et al. [7] used the CAR model to account for spatial dependence among the locations of drug abuse reporting centers. Zou et al. [8] proposed to accommodate spatio‐temporal variations in syndromic surveillance using a Bayesian conditional probabilistic approach. Heaton et al. [9] applied a similar absorbing state model to influenza/pneumonia fatality data. Similar model‐based approaches have been considered in Knorr‐Held and Richardson [10], Martínez‐Beneito et al. [11], and Zhou and Lawson [12].

In this paper, we focus on the early and accurate detection of outbreaks of diseases, which could be either contagious or noncontagious. In syndromic surveillance, there is no definitive diagnosis of an outbreak at the early stage. Our methodology has been created exclusively to detect disease outbreak early, to monitor the spatio‐temporal spread of an outbreak, and to provide decision supporting tools for immediate analysis and feedback to public health authorities. This approach will speed up the decision making process and the implementation of countermeasure procedures.

We propose using a flexible hierarchical Bayesian model to partition the variability and quantify uncertainties in a unified framework. Our model can accommodate both spatial effects and temporal dynamics. It also assumes that the spatial aspects arise from a nonseparable spatio‐temporal conditional autoregressive (STCAR) model, where the temporal aspect is a direct result of a plausible Markov structure. We introduce a rigorous, probabilistic, epidemiological model to explicitly account for the disease dynamics based on human contact, and other exogenous variables such as local population.

Our hierarchical model decomposes the source variabilities into different components, which have reasonable epidemiological interpretations. Numerical results suggest that the model performs sensibly and is robust to various less than ideal settings and conditions. In a companion study, we are conducting sensitivity analysis with respect to signal‐to‐noise ratio (SNR), choice of priors, missing and superfluous edges in the network structure, and other possible model misspecifications. We also considered a particular model misspecification when the underlying true model has a dynamic Susceptible‐Infected‐Recovered (SIR) structure (See e.g., Keeling and Rohani [13]). We have demonstrated that accounting for spatio‐temporal correlation improves assessing the impact of outbreak distributions, produces accurate maps of occurrence, and allows for good prediction performance.

In this paper we illustrate our methodology using data from Indiana Public Health Emergency Surveillance System (PHESS). The data set is based on emergency department (ED) visits for influenza‐like illness (ILI) and respiratory illness over the three‐year period 2008–2010. Besides the usual methodology issues described in the next section, two major challenges were encountered. The first involved masking of the day of the week for confidentiality reasons and the second was the effect of choice of the network to avoid zero counts and provide more accurate results. Details are provided in the next section. This paper has several innovative features compared to previous studies as in Zou et al. [8] and Heaton et al. [9], since we have incorporated the Day of Week effect, the different network structures and decision rules.

This paper is organized as follows. In Section Methods, we introduce a spatio‐temporal methodology for syndromic surveillance, and describe some properties of the model. In Section Results and discussion, we present some numerical studies and results on a real surveillance data set. We also illustrate how one decision‐making framework behaves when it is applied to the output of our model in Section An illustration of the trade‐off between false positives and timeliness of detection. Finally, in Section Conclusions, we give a conclusion and discuss possible improvements of our current methods and future research directions.

## Methods

In this paper, we mainly focus on changes happening in discrete time and on contagious diseases. The basic model described in Zou et al. [8] is adopted here. Specifically, let *Y*_*i*_(*t*) be the number of individuals with a specific syndrome recorded at site *i* on day *t*, where $i=1,\dots, m$ and $t=1, \dots,T$. We assume that when a disease outbreak occurs, *both the level and the spatio‐temporal structure* of *Y*_*i*_(*t*) change.

### Basic model

We model the number of counts *Y*_*i*_(*t*) by a Bayesian hierarchical model. We assume the first stage is Poisson with canonical link (log linear), so that in the absence of an epidemic, the mean function of the Poisson count at location *i* is *μ*_*i*_(*t*). When there is an epidemic, a second component is added to the baseline. The additional intensity in epidemic state is represented by *λ*_*i*_(*t*). We use an indicator function *δ*_*i*_(*t*) as the mark of whether an epidemic is present. Thus, conditional on *μ*_*i*_(*t*),*λ*_*i*_(*t*) and *δ*_*i*_(*t*), the first stage model becomes

(1) $$ {\small{\begin{aligned}  Y_{i}(t) \sim \text{Pois}\left(\mu_{i}(t)+ \delta_{i}(t)\lambda_{i}(t)\right), \, \mathrm{independently,} \, i=1,\cdots,m. \end{aligned}}}  $$

Let ***μ***(*t*)=(*μ*_1_(*t*),⋯,*μ*_*m*_(*t*))^*T*^,***λ***(*t*)=(*λ*_1_(*t*),⋯,*λ*_*m*_(*t*))^*T*^ and ***δ***(*t*)=(*δ*_1_(*t*),⋯,*δ*_*m*_(*t*))^*T*^. We assume ***μ***(*t*),***λ***(*t*) and ***δ***(*t*) are mutually independent.

#### Model for ***μ***(*t*)

Let $\theta _{i}(t) = \log (\mu _{i}(t))$. We assume that $\theta _{i}(t)=\boldsymbol {X}_{i}^{T}(t){\boldsymbol {\beta }}_{\mu }+\varepsilon _{i}(t)$, where $\boldsymbol {X}_{i}(t)=(1, X_{i, 1}(t), \cdots, X_{i, p}(t))^{T},\, i=1, \dots, m$, represent covariates such as population size, ***β***_*μ*_=(*β*_*μ*_,0,*β*_*μ*_,1,⋯,*β*_*μ*_,*p*)^*T*^ are regression coefficients, and $\varepsilon _{i}(t) \sim N\left (0,\sigma _{\mu }^{2}\right), i= 1, \cdots, m,$ are independently and identically distributed. Spatial and temporal variations can be incorporated in the covariates.

#### Model for ***λ***(*t*)

When there is an outbreak, we presume that the additional intensity *λ*_*i*_(*t*) follows a model with spatio‐temporal conditional autoregressive (STCAR) structure. Specifically, let $\eta _{i}(t)=\log (\lambda _{i}(t))$; then,

$$\eta_{i}(t) = {\boldsymbol{U}}_{i}^{T}(t){\boldsymbol{\beta}}_{\lambda}+\xi_{i}(t), $$

where $\boldsymbol {U}_{i}(t)=(1, U_{i, 1}(t), \cdots, U_{i, p}(t))^{T},\, i=1, \dots, m$, can be epidemic‐specific covariates, and ***β***_*λ*_=(*β*_*λ*,0_,*β*_*λ*_,1,⋯,*β*_*λ*_,*q*)^*T*^ are covariate coefficients. We assume that the first column of ***U***_*i*_(*t*) consists entirely of ones, in which case *β*_*λ*_,0 becomes a scaling factor that can be interpreted as the relative size of the outbreak compared to the baseline. Spatial relationships between sites are represented by an adjacency matrix *W*=(*w*_*ij*_): if sites *i* and *j* are adjacent, then *w*_*ij*_=1, and otherwise *w*_*ij*_=0. Also, by convention, *w*_*ii*_=0. The *ξ*_*i*_(*t*) are stipulated to satisfy

(2) $$ \begin{aligned} \xi_{i}(t)|\boldsymbol{\xi}_{-i}(t),\boldsymbol{\xi}(t-1) \sim N & \left(\frac{\rho_{1}}{w_{i+}} \sum_{j}w_{ij}\xi_{j}(t) \right.\\ &\left.\quad +\, \rho_{2} \xi_{i}(t-1), \frac{\sigma_{\lambda}^{2}}{w_{i+}}\right),  \end{aligned}  $$

where $w_{i+}=\sum _{j}w_{\textit {ij}}$. In (2), ***ξ***(*t*)=(*ξ*_1_(*t*),⋯,*ξ*_*m*_(*t*))^*T*^, and ***ξ***_−*i*_(*t*) is the vector ***ξ***(*t*) excluding the *i*th component. Here *ρ*_1_ is a spatial correlation and *ρ*_2_ is a temporal correlation parameter. We take ***ξ***(1)=(0,⋯,0)^*T*^ as the initial values at *t*=1.

#### Model for ***δ***(*t*)

Let *δ*_*i*_(*t*)=1 if the disease is present at site *i* on day *t* and *δ*_*i*_(*t*)=0 otherwise. Currently, we employ an *absorbing state model* for ***δ***: (3)

where *N*_*i*_ is the set of spatial neighbors of *i*, that is, *N*_*i*_={*j*:*w*_*ij*_=1}. We assume that the *δ*_*i*_(*t*+1) are conditionally independent given ***δ***(*t*). The two parameters in (3) have straightforward interpretations: *p*_*s*_ is spontaneous generation rate for outbreaks, i.e., the probability of an outbreak when neither the site nor any of its neighbors has an outbreak, and *p*_*c*_ is contagion rate for transfer of outbreaks at neighbors to a site without an outbreak. *τ*_*i*_ is the population in site *i*. In this formulation, we can incorporate spatial heterogeneity and disease transmission due to the population effect. It is analogous to the transmission mechanism in the SIR model, where the transmission rate is proportional to the product of populations at two sites.

Since we assume the three components *μ*_*i*_(*t*), *δ*_*i*_(*t*) and *λ*_*i*_(*t*) are mutually independent, then {*Y*_*i*_(*t*);*t*≥1} has the same distribution as

(4) $$ Y_{i}(t) = Y_{i}^{\mu}(t) + \delta_{i}(t)Y_{i}^{\lambda}(t),  $$

where $Y_{i}^{\mu }(t) \sim \text {Pois}(\mu _{i}(t))$, $Y_{i}^{\lambda }(t) \sim \text {Pois}(\lambda _{i}(t)),$ and $Y_{i}^{\mu }(t)$, *δ*_*i*_(*t*) and $Y_{i}^{\lambda }(t)$ are conditionally independent given ***λ*** and ***μ***. The components of this decomposition reveal insights into the variability of the observed data. In spite of the seemingly simple count data structure, the model can lead to rapid and accurate detection methods for disease outbreak.

### The day of week effect

There are some challenging issues involved in this data set. Health Insurance Portability and Accountability Act (HIPAA) regulations require significant confidentiality, therefore the data are supplied in a very controlled fashion. Initially the data were provided with perturbed dates of the ED visits to preserve confidentiality and share‐ability. Such perturbing smoothes the daily ILI and respiratory illness counts, which attenuates the signal of the start of an illness outbreak. This makes correctly predicting the start of the outbreak much more difficult, if not impossible.

In addition, perturbing the day of ED visit leads to a uniform distribution of the counts over the seven‐day week. This contradicts the DoW effect that is prominent in ED visits [14]. Hafen et al. [15] also document that the distribution of ED visits varies according to the day of the week. For example, the average number of visits on Monday is significantly greater than the average visits over the other days of the week. The day of the week regularity is not limited to only the hospital data, but is also present in other patient care facilities.

Therefore, we requested and were granted a new data set with correct dates to warrant a valid and accurate analysis. (Of course, extra safety measures were taken to preserve confidentiality). We conducted simulations on date perturbation and confirmed that it removes the day of effect pattern from the distribution.

The analysis reported in Section Results and discussion is of *daily reports* of ILI and respiratory syndrome at hospital emergency departments. The actual disease process is latent, and is not directly observable. From this real data set, we observe that ED reports have a pronounced day‐of‐week effect: reports are high on Sundays, when other facilities, such as urgent care centers, are not available, and low in the middle of the work week. Therefore, it becomes apparent that it is necessary to account for the DoW effect in syndromic surveillance.

According to the literature, there are several possible methods to represent a DoW effect. For example, one can adopt the indicator (dummy) variable approach. It is easy to implement but may not be flexible enough to capture other complicated patterns. One can also apply other more complicated seasonal ARIMA models as in Box et al. [16].

The methods with time series roots, such as trigonometric functions or ARIMA models, are not well‐suited to our modeling structure. Instead, we employ a day of week indicator covariate. We mainly focus on the multiplicative day of week effect on the two components *μ* and *λ*. That is, we include additive terms $\boldsymbol {X}_{\text {DoW}}^{T} \beta _{\mu }, \text {DoW}$ and $\boldsymbol {X}_{\text {DoW}}^{T} \beta _{\lambda }, \text {DoW}$ in the expression of $\log (\mu)$ and $\log (\lambda)$. So the full model becomes

(5) $$ \begin{aligned}  \mu_{i}(t) =&\,\exp\left(X_{\mu}^{T}(t)\boldsymbol{\beta}_{\mu} + \boldsymbol{X}^{T}_{\text{DoW}}\beta_{\mu}, \text{DoW}+ \varepsilon_{i}(t)\right);\\ &\hspace*{3pt}\varepsilon_{i}(t) \sim N\left(0,\sigma_{\mu}^{2}\right), \end{aligned}  $$

(6) $$ \begin{aligned} \lambda_{i}(t)=\exp\left(X_{\lambda}^{T}(t)\boldsymbol{\beta}_{\lambda} + \boldsymbol{X}^{T}_{\text{DoW}}\beta_{\lambda, \text{DoW}}+ \xi_{i}(t)\right), \end{aligned}  $$

where *β*_*μ*_,DoW and *β*_*λ*_,DoW quantify the multiplicative day of week effects in the syndrome counts, ***X***_DoW_=(1,1,0,0,0,0,0)^*T*^. This suggests that the DoW effects are configured as 1 for Sunday and Monday, and 0 for Tuesday through Saturday.

### Some comments on the absorbing state model

Note that the absorbing state model (3) emphasizes the ability of early detection of outbreaks, but is not designed to predict the end of an epidemic. Surveillance data must be disseminated quickly to public health practitioners and decision makers. The more quickly outbreaks can be detected, the more effectively a public health agency’s intervention and disease control programs can prevent further morbidity or mortality. For example, an anthrax outbreak occurred in the Fall 2001, and was identified by a clinician and immediately reported to public health officials. This led to a prompt reaction to treatment of exposed individuals and informing the general public (CDC 2001). It becomes critical that the main purpose of a surveillance method lies in its timeliness and effectiveness of detecting new outbreaks or epidemics. Public health surveillance data can also provide information about when a disease outbreak fades out and ends. This information could result in saving public health resources and create less anxiety in the general public.

This research was approved by the Indiana University Institutional Review Board (IRB), Protocol Number 1011003359. Access to the relevant de‐identified patient data was approved by the Indiana Network for Patient Care (INPC) Management Committee.

## Results and discussion

In this section, we will present results regarding a real data application of the analysis of the 2008–2010 Indiana respiratory syndrome counts.

### Data description

Our data set is derived from emergency department visits for Influenza‐like illness and respiratory illness in the Indiana Public Health Emergency Surveillance System (PHESS) [17]. The system integrates data flows from a network of hospitals across Indiana for use in public health disease surveillance and clinical research. Started in 2004, the network has grown to include over 110 hospitals covering more than 90% of ED visits in Indiana. Advances in electronic medical record systems and health information exchange are refocusing public health efforts toward greater use of information systems to automate disease surveillance. Indiana hosts the Indiana Network for Patient Care (INPC), the largest and longest‐running health information exchange (HIE) in the U.S. Observational clinical data gathered by the INPC primarily supports clinical care processes, and are also repurposed to support public health initiatives such asPHESS.

For the last several years, the PHESS system has received real‐time data from participating hospitals, accumulating more than 2 million transactions per year, and has aided detection of public health outbreaks including gastrointestinal illness and carbon monoxide poisoning. It also supports monitoring of influenza and other diseases at the population level. The system’s ability to track data from physician offices and medical facilities across Indiana provides public health officials with early warning of outbreaks of influenza and other communicable diseases in Indiana. It can support identification of weather‐related health conditions or food‐borne illnesses, enabling more timely actions including the alerting of appropriate medical personnel and policymakers. Public health authorities may identify outbreaks more rapidly than they could before by employing the unique capabilities of the system to securely exchange health information when and were it is needed. It is crucial to detect events quickly so that they can respond early enough to intervene and prevent greater disease spread [18].

ED visit data are collected in near real‐time and are transmitted to the PHESS system within minutes of actual visits. For this study, the variables in the analysis data set include date of visit, patient age, gender and residence ZIP code. Further, the patient’s free‐text chief complaint is categorized using a naive Bayes classifier from the University of Pittsburgh’s Real‐time Outbreak and Disease Surveillance (RODS) laboratory [19].

However, there are several difficulties in analyzing these real surveillance data. First, the data streams contain complex dependency structures in space and time. Second, different classification rules convert patient chief complaints into different syndromes. We adapted the classification rules from the RODS project to convert patient chief complaints into different syndromes. The mapping is many‐to‐many. For example, a patient could have both “influenza” and “respiratory” syndromes. Third, the Indiana State Department of Health employs existing surveillance methods such as the CDC’s Electronic Surveillance System for the Early Notification of Community‐Based Epidemics (ESSENCE) system, which may generate many false alarms due to multiple comparison issues. As a result, the routine unfiltered ESSENCE results may be of limited value for daily operational purposes. Finally, data perturbations protecting patient privacy and confidentiality are often needed (and were implemented in this analysis) in order to produce sharable results while still maintain the same validity and credibility.

**The Choice of the Network.**It is interesting to note that daily syndrome counts at county level create problems with zeros, especially in the summer months where regular influenza activity is low. This could cause unstable inference results and additional model complexity.

**Figure 1:**
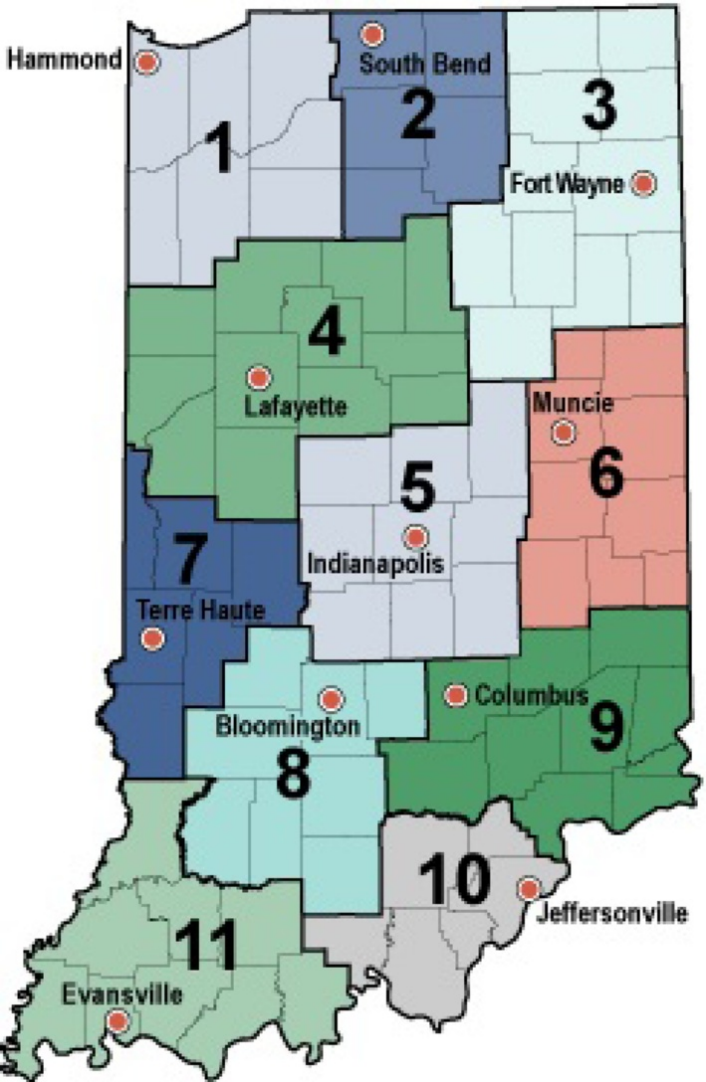
**Indiana state map with 11 regional labor markets.**

One can proceed with a zero inflated model where a point mass is added at zero in order to accommodate the excessive zeros in the observations. However, this would pose additional complexity in the model and potentially make the computing slow. Therefore, in this empirical study, we opt to aggregate to a network that contains 11 regional labor markets in Indiana, which reflects one primary transmission path for most commutable diseases and is also considered a viable surveillance network by the public health agencies. By applying our methodology on the alternative aggregated spatial structure, we not only eliminate the zero counts issues, but also are able to borrow additional information from other economic and workforce indicators for surveillance purposes. We further comment on how surveillance might be improved by broadening the spatial region, while still assuring validity and consistency. Figure 1 shows the Indiana map divided into eleven regional labor markets. The most populated area is the Indianapolis greater metropolitan area in central Indiana, which contains the state capital and many of the state’s largest employers.

This was the first meaningful confidentiality issue we faced with the data that we were initially provided. We applied a simple date perturbation by adding random ± 1,2,3 days to the real date variable, which made the distribution of days of the week uniform and completely eliminated the day of week effect, and affected the accuracy of detecting the start of an outbreak. We stress that there are many interesting data confidentiality issues in terms of public health surveillance records with spatial and temporal characteristics. Ensuring privacy and security of health information, including information in electronic health records, is the key component to building a successful syndromic surveillance system.

### Implementation

The PHESS data set for this study contains over seven million observations for ILI and respiratory syndrome and classification counts from ED visits through the three‐year period between 2008 and 2010. The date of visit variable contains information of exact date and time of the actual visit. However, for confidentiality and simplicity, we use only daily counts in this analysis, which comprises of 1095 days of data. The PHESS data are rich in the sense that they not only have valuable individual information such as patients’ residence zip code, gender and age, but also contain different data streams including daily counts of ICD‐9 code, patient chief complaints.

The covariate included in this analysis is the population size based on the 2010 national census data for Indiana. The model is then completed with the prior specifications for the hyperparameters (***β***_*μ*_,***β***_*λ*_,*σ*_*μ*_,*σ*_*λ*_,*ρ*_1_,*ρ*_2_,*p*_*s*_,*p*_*c*_). Here we take diffuse priors on the covariate coefficient parameters, inverse gamma priors on the variance parameters, uniform priors restricted to the interval (‐1, 1) for *ρ*_1_ and *ρ*_2_, and log normal priors restricted to the interval (0, 1) for *p*_*s*_ and *p*_*c*_. The choices of hyperparameters represent vague prior information and ensure posterior propriety. The choices of priors are considered to be stable and robust to misspecifications in our simulation studies (not reported here). To improve Markov chain Monte Carlo(MCMC) convergence and model inference, we also use the previous one‐year data to inform the following year’s prior distribution parameters.

While the methodology itself is rather involved, being based on the theory of Gaussian Markov random fields, the actual computations are reasonably fast. All computations were carried out in the open source statistical software package R on a Windows desktop. The computations related to period I and III in Section A surveillance case study took approximately 4 hours per time period to fit the model, estimate the parameters and compute the real‐time probabilities. Running the MCMC simulations and computing the probability estimates for period II took about 9 hours. This makes our approach potentially useful for daily surveillance purposes.

### A surveillance case study

In this section, we present a three‐part case study for surveillance with distinctive features and interesting findings. The data are daily respiratory syndrome counts based on the Indiana PHESS system definition. Cases were emergency department visits for respiratory illness from the whole state of Indiana over a three‐year period from January 1, 2008 to December 31, 2010. We looked at three time periods that had definite outbreaks confirmed via retrospective analysis by public health domain experts. The three periods are spring 2008, fall 2009 and fall 2010, i.e., January 1, 2008–March 31, 2008, June 1, 2009–December 31, 2009, and August 1, 2010–October 31, 2010. The results are listed in the following.

**Figure 2:**
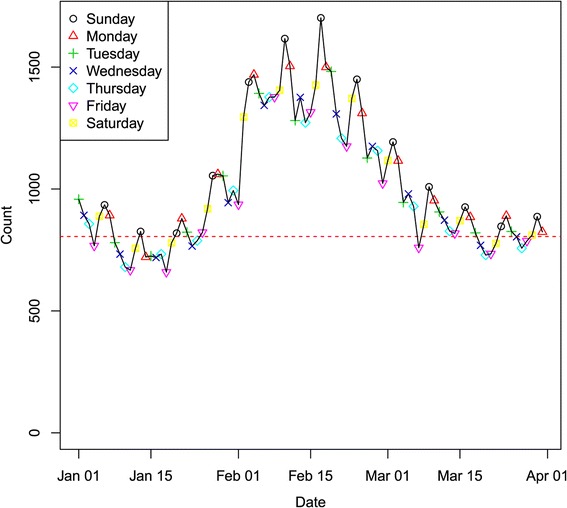
**Respiratory syndrome data stream for the whole state in spring 2008.**

**Period I:** Only a very limited training data set is available for the first period to test the method; namely, the first two weeks of data, i.e., January 1–14, 2008, could be used. We ran the detection model for the three‐month period. Figure 2 illustrates the overall state total counts for respiratory syndromes over the three‐month period. The daily aggregated syndrome counts are plotted for the whole state with day of week symbols superimposed to highlight the weekly pattern. In the plot, the DoW effect is clearly evident. Most of the high counts occur at either Sunday or Monday, while counts are usually low on Fridays.

**Figure 3:**
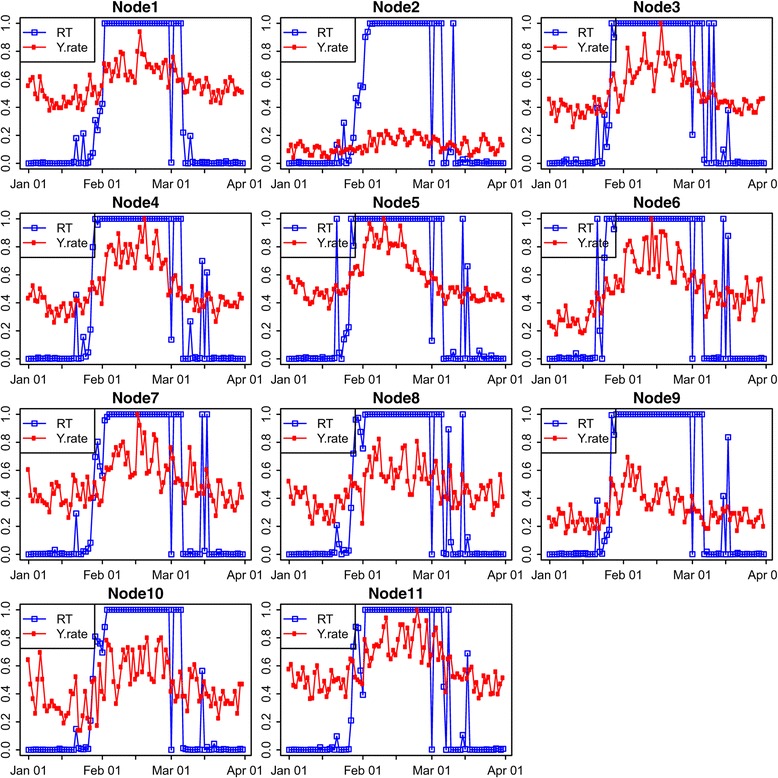
**Real‐Time (** ***P*** **(** ***δ*** **(** ***t*** **)=1|** ***Y***_**1:*t***_ **)) probabilities for each individual region in spring 2008, where dates are represented as R** ***mmdd*** **, e.g, R0113 stands for the real‐time probability on January 13.**

The model inference was then performed with DoW effect on this data set as in Models (1)–(6). Figure 3 demonstrates the model inference results for each individual region over the time course. We ran MCMC using all the data up to the current time *t* for each day to determine the posterior. The real‐time posterior probability that ***δ***(*t*)=1 (RT) is plotted for each node with the incidence rate (y.rate) scaled and superimposed on the same graph. This can highlight the fact that our model is robust to low signal (low incidence rates at some nodes) with high precision (timely detection and low false alarms even for sites with very low incidence rates).

**Figure 4:**
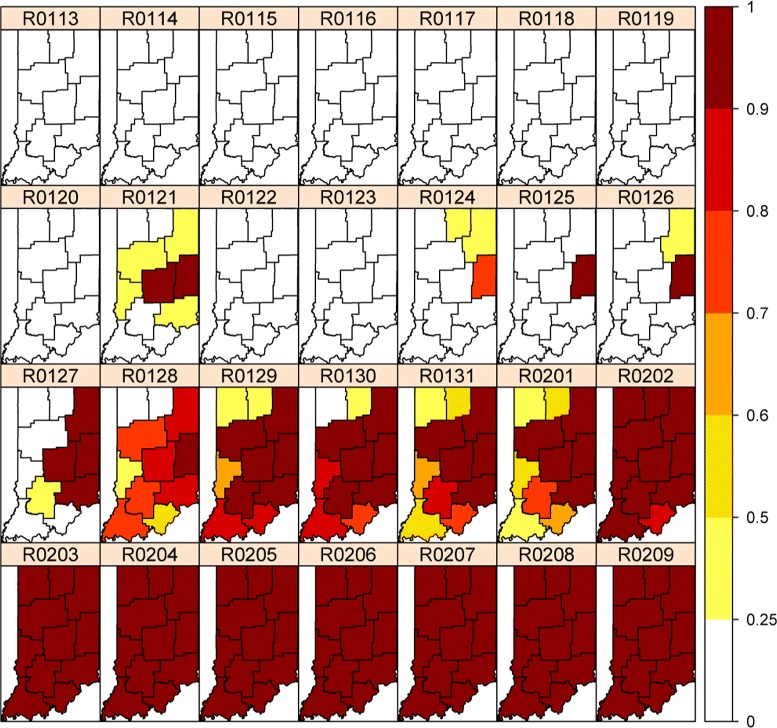
**Real‐Time (** ***P*** **(** ***δ*** **(** ***t*** **)=1|** ***Y***_**1:*t***_ **)) probabilities for a four‐week period in spring 2008.**

Figure 4 shows the spatial and temporal dynamics of the outbreak evolution from our model inference. The total counts for the state in Figure 2 indicate that the outbreak occurred around February 1. Figure 4 indicates that the possible outbreak starts in Regions 5 and 6 on January 21, though this appears to be a false positive. This is also confirmed in Figure 3 for nodes 5 and 6 which demonstrates that the outbreak probably started in node 6 on January 24 or January 25 and then spread to nodes 3, 5 and 9 on January 27. This also indicates that the slightly elevated counts in Figure 2 for January 26–31 are due to an outbreak in the state, but are so subtle for January 26 and January 27 that they could not be called without the more detailed spatio‐temporal analysis in Figures 3 and 4.

Period I is very challenging due to the limited information from the training data, however, our method works reasonably well and is able to show the spatio‐temporal evolution with uncertainty measures associated with each location and time point.

**Figure 5:**
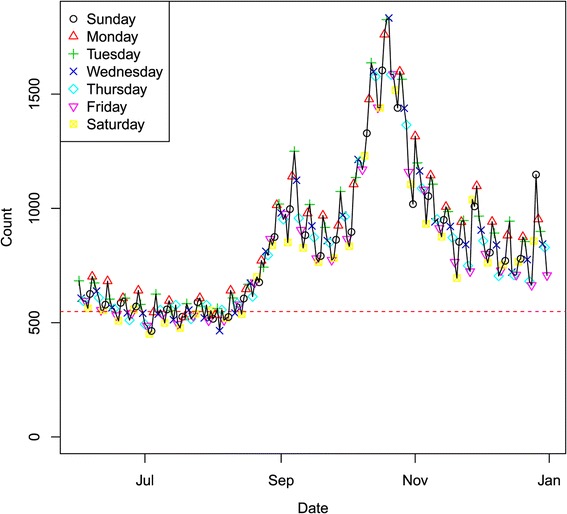
**Respiratory syndrome data stream for the whole state in fall 2009.**

**Figure 6:**
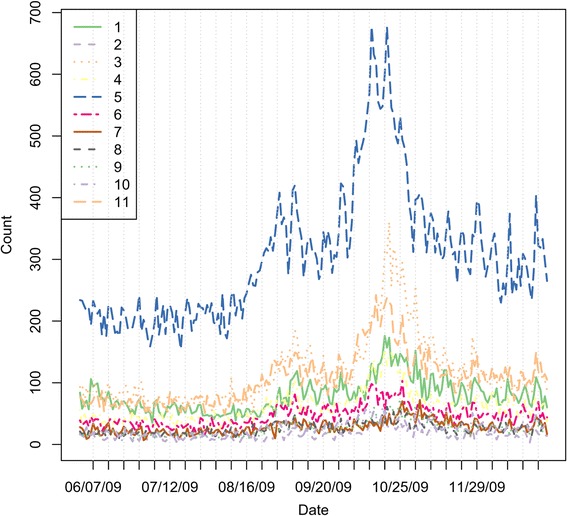
**Respiratory syndrome data stream for individual regions in fall 2009, where dates are represented as Y** ***mmdd*** **, e.g, Y0809 stands for the real‐time probability on August 09.**

**Period II:** For the second period, we looked at the seven‐month period in 2009, June 1–December 31, 2009. This is when the H1N1 outbreak occurred. As shown in Figure 5, there is an unusual high peak in mid/late October due to the large‐scale outbreak of H1N1 in the fall season. However, the increasing trend started well before the peak, which occurred around mid/late August. To better appreciate the difference due to spatial heterogeneity of the H1N1 outbreak, Figure 6 demonstrates the evolution of the outbreak over time for each individual region. We can see that by only observing the time series of the syndrome counts, some regions have a prominent outbreak pattern while others show little to no sign of severe activity. This can be misleading to public health officials.

**Figure 7:**
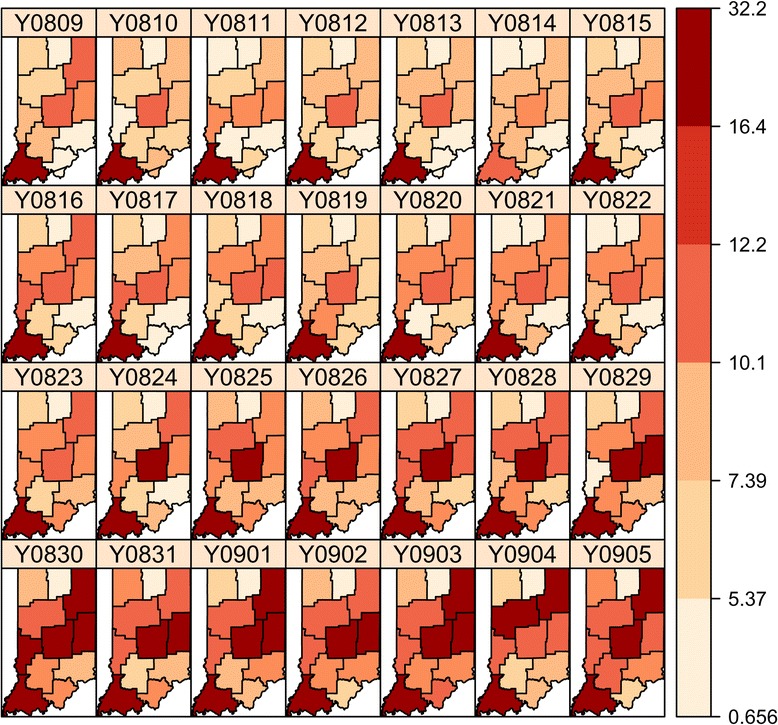
**Rates of respiratory syndrome per 100,000 population for a four‐week period in fall 2009.**

**Figure 8:**
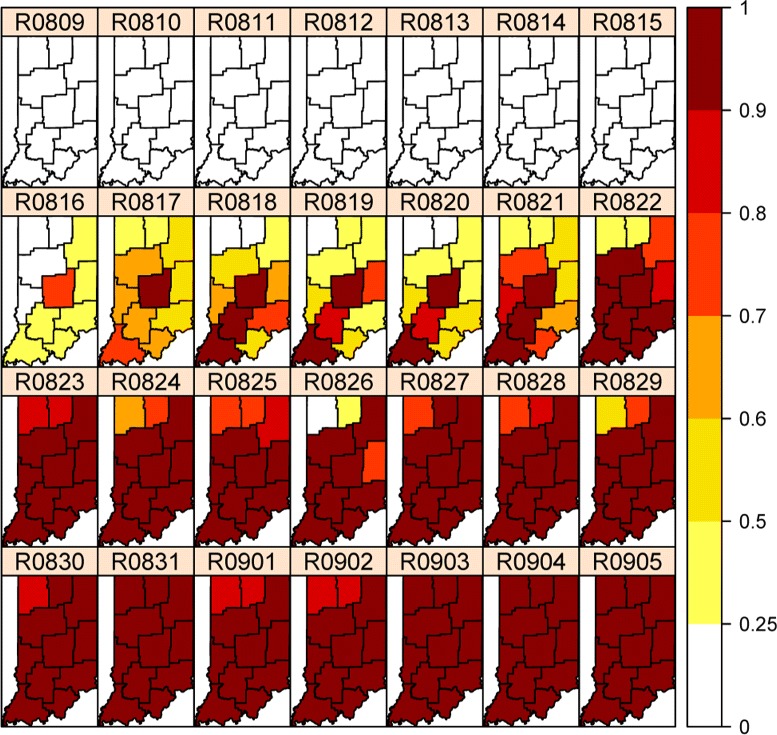
**Real‐Time (** ***P(δ***(***t***)=1|***Y***_1:***t***_ **)** **) probabilities for a four‐week period in fall 2009.**

By taking into account the spatial and temporal correlation among the regions, we analyze the data using the background level based on the summer average counts between June and August. From the incidence rate in Figure 7, we see that regions with low population have the highest rates and are misleading visually. However, our model can successfully detect the outbreak starting at the Indianapolis metropolitan area in a timely fashion with high precision, as shown in Figure 8. It turns out that the outbreak first started in the Indianapolis metropolitan at August 16. In the next two days it spread to neighboring regions Bloomington and its neighbor Evansville area. The outbreak developed into a state‐wide epidemic in just a couple of days.

If the public health agencies relied on monitoring just the overall state‐wise syndrome counts or the individual county‐wise incidence rate plot, they would miss the early start of the epidemic. Consequently, they would miss the most effective period of intervention, which would result in significant costs in public health.

**Figure 9:**
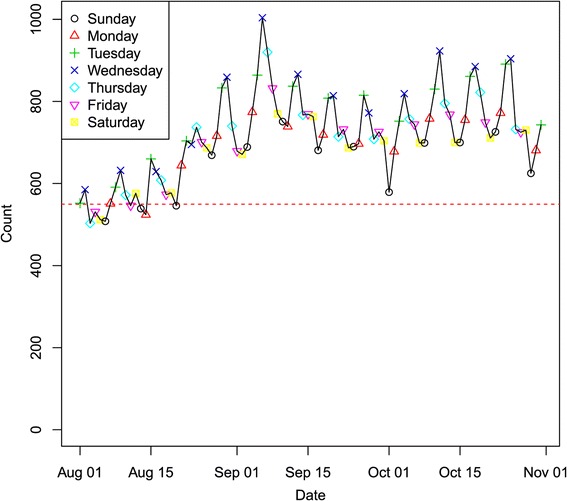
**Respiratory syndrome data stream for the whole state in fall 2010.**

**Figure 10:**
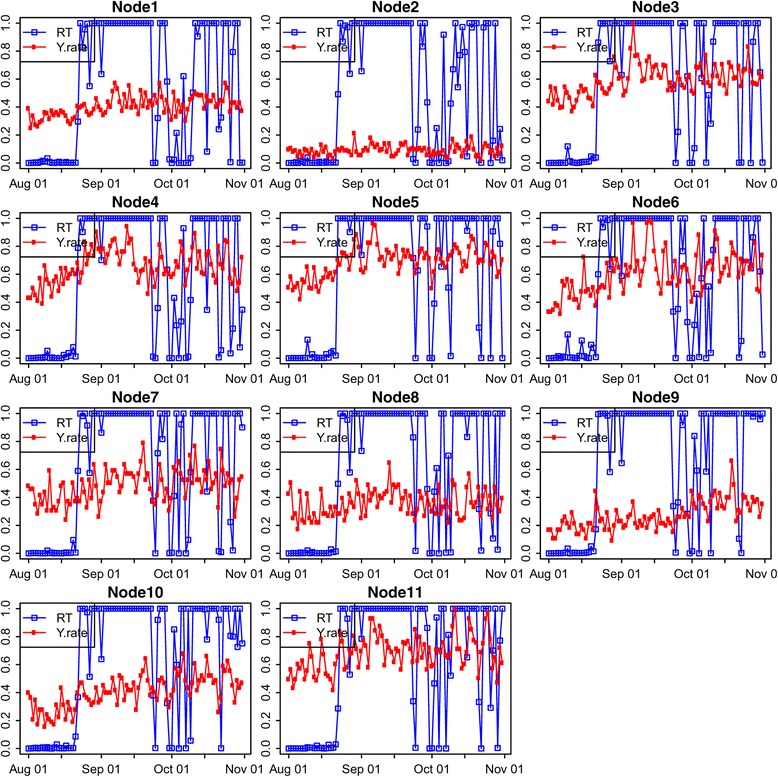
**Real‐Time (** ***P*** **(** ***δ*** **(** ***t*** **)=1|** ***Y***_**1:*t***_ **)) probabilities for each individual region in fall 2010.**

**Figure 11:**
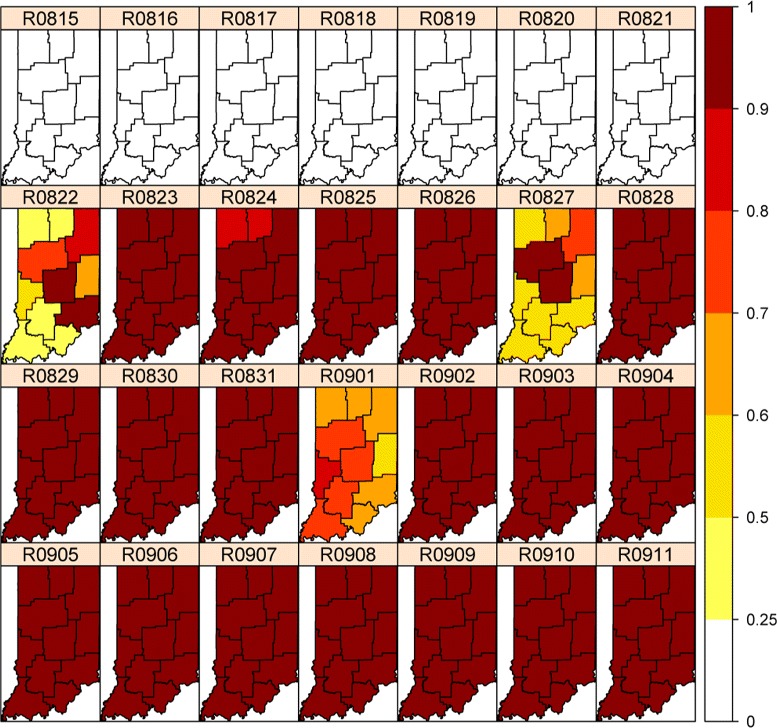
**Real‐Time (** ***P*** **(** ***δ*** **(** ***t*** **)=1|** ***Y***_**1:*t***_ **)) probabilities for a four‐week period in fall 2010.**

**Period III:** For the third case, we look at the time period of August 1–October 31, 2010. Figure 9 shows the overall state total counts for respiratory syndromes over the three‐month period. This case is interesting because in the year following the H1N1 outbreak, Indiana had a very mild fall flu season compared to previous years. Therefore, timely and effective outbreak detection is quite challenging due to low signals compared to the background. Figure 10 shows the results of the real‐time probability for each individual region. We note that the model is still able to detect the outbreak in a timely fashion. In Figure 11, we can see that the outbreak started in the Indianapolis metropolitan area and Columbus area simultaneously, then it quickly spread to other regions in the following days.

This case study shows that our methodology can, indeed, yield satisfactory surveillance performance in an applied setting. Here we emphasize timeliness and apply real‐time analysis and visualization tools to syndrome (not actual diagnosis) data in electronic form so as to detect unexpected patterns that warrant investigation. The lead‐time our method provides is crucial to public health authorities to take more effective public health actions. In this study, while we focus on maps showing spatial and temporal dynamics of disease outbreaks, the Bayesian posterior distributions contain much more information such as uncertainty measures and predictive probabilities for the outbreaks. For example, the posterior mean estimate of the *p*_*c*_ parameter for period II is 0.9047, which is consistent with the rapid spread pattern shown in Figure 8.

### An illustration of the trade‐off between false positives and timeliness of detection

For syndromic surveillance systems, another valuable tool for public health authorities is to provide some decision rules under different scenarios. The ideal solution is quickest detection of outbreaks with as few false alarms as possible. The first of these is important because failure to detect means failure to act. On the other hand, frequent false alarms are expensive and lead to distrust of the system by the public. Of course, these two goals are incompatible, and ultimately decision makers must make tradeoffs.

One note about false positives is that it is very common to have false alarms when the study involves a large spatial region and a long time period. Existing methods such as spatial scan statistics require multiple hypothesis tests. Thus, one needs to control the false alarms very carefully. As an illustration, we set up decision rules such that for a given threshold *ϖ*, the alarm is sounded when the probabilities exceed the threshold for *N* consecutive days. For example, if *N*=2, then we declare a start of outbreak at $\min \{t: P(\boldsymbol {\delta }(t) = 1 |Y_{1:t}) > \varpi \; \text {and} \; P(\boldsymbol {\delta }(t+1) = 1 |Y_{1:t+1}) > \varpi \}$. This way, we can limit false positives while still detecting abnormal patterns early and accurately.

As a form of illustration for the impact of different decision rules on the performance of our surveillance methodology, we analyzed the PHESS data using the aforementioned decision rule with different threshold *ϖ* and number of days *N* for the same three time periods as in Section Results and discussion. Results are reported in Tables 1, 2 and 3. These tables contain declared epidemic start dates based on the decision rules that the posterior probability of *P*(*δ*(*t*)=1|*Y*_1:*t*_)>*ϖ* for *N* consecutive days. In order to show the trade‐off between early detection and false alarms heuristically, we choose *ϖ*=0.2,0.5,0.8 and *N*=1,2,3 respectively. As illustrated in Table 1, if one should select *N*=1, the system becomes very sensitive and declares the ILI epidemic start at Region 5 on 01/21 in 2008, regardless of what the threshold *ϖ* is. However, if we increase to *N*=2, the alarm is not sounded until 01/26 for *ϖ*=0.2 and 01/27 for *ϖ*=0.5,0.8, respectively. This suggests that the declaration on 01/21 could be a potential false positive. Similarly, in 2009, the model declares a start of epidemic at Node 10 on 08/16 for *ϖ*=0.2, 08/17 for *ϖ*=0.5 and 08/22 for *ϖ*=0.8, no matter what *N* is. Since *ϖ*=0.2 is very low, 08/16 may be a potential false positive. Nevertheless, the public health agencies have to decide the trade‐off between the two other thresholds. If *ϖ*=0.5 is chosen, then they would gain five days of time to verify the cases and evaluate different courses of intervention and prevention measures. As the threshold goes from *ϖ*=0.5 to *ϖ*=0.8, we get rid of some potential false positives, but if the outbreak did happen on 08/17, we lose five days and potentially cause huge loss in morbidity and mortality. We were pleasantly surprised that our model performs almost ideally without any ambiguity in 2010, since the declared start date are very consistent with different threshold and number of consecutive days. It will further facilitate quick dissemination of the findings to those who need to know, and rapid decisions on proper course of actions can be made by health care agencies.

**Table 1:** **Declared start of ILI epidemic in 2008 for each region, under various decision rules**

**Decision rule**		**Declared start of epidemic in region**										
**Days** ***N***	**Threshold** ***ϖ***	**1**	**2**	**3**	**4**	**5**	**6**	**7**	**8**	**9**	**10**	**11**
1	.2	1/24	1/24	1/21	1/21	1/21*	1/21	1/21	1/21	1/21	1/27	1/27
1	.5	2/02	1/31	1/27	1/28	1/21*	1/21	1/29	1/28	1/27	1/28	1/28
1	.8	2/02	2/02	1/27	1/29	1/21*	1/21	1/30	1/29	1/27	1/29	1/29
2	.2	1/29	1/29	1/26	1/27	1/26	1/21	1/28	1/27	1/27	1/27	1/27
2	.5	2/02	1/31	1/27	1/28	1/27	1/24	1/29	1/28	1/27	1/28	1/28
2	.8	2/02	2/02	1/27	1/29	1/27	1/25	2/02	1/29	1/27	2/02	1/29
3	.2	1/29	1/29	1/26	1/27	1/26	1/24	1/28	1/27	1/27	1/27	1/27
3	.5	2/02	1/31	1/27	1/28	1/27	1/24	1/29	1/28	1/27	1/28	1/28
3	.8	2/02	2/02	1/27	1/29	1/27	1/25	2/02	1/29	1/27	2/02	2/02

The epidemic is declared when *P*(*δ*(*t*) = 1*Y*_1:*t*_) exceeds the threshold *ϖ* for *N* consecutive days. “*” represents a case of potential false alarm.

**Table 2:** **Declared start of ILI epidemic in 2009 for each region, under various decision rules**

**Decision rule**		**Declared start of epidemic in region**										
**Days** ***N***	**Threshold** ***ϖ***	**1**	**2**	**3**	**4**	**5**	**6**	**7**	**8**	**9**	**10**	**11**
1	.2	8/17	8/17	8/16	8/17	8/16	8/16	8/16	8/16	8/16	8/16*	8/16
1	.5	8/23	8/23	8/17	8/17	8/16	8/17	8/17	8/17	8/17	8/17*	8/17
1	.8	8/23	8/23	8/23	8/22	8/17	8/22	8/21	8/18	8/22	8/22	8/18
2	.2	8/21	8/21	8/16	8/17	8/16	8/16	8/16	8/16	8/16	8/16*	8/16
2	.5	8/23	8/23	8/21	8/17	8/16	8/17	8/17	8/17	8/17	8/17*	8/17
2	.8	8/30	8/27	8/23	8/22	8/17	8/22	8/21	8/18	8/22	8/22	8/18
3	.2	8/21	8/21	8/16	8/17	8/16	8/16	8/16	8/16	8/16	8/16*	8/16
3	.5	8/23	8/23	8/21	8/21	8/16	8/17	8/17	8/17	8/20	8/17*	8/17
3	.8	8/30	8/30	8/23	8/22	8/17	8/22	8/21	8/18	8/22	8/22	8/18

The epidemic is declared when *P*(*δ*(*t*) = 1*Y*_1:*t*_) exceeds the threshold *ϖ* for *N* consecutive days. “*” represents a case of potential false alarm.

**Table 3:** **Declared start of ILI epidemic in 2010 for each region, under various decision rules**

**Decision rule**		**Declared start of epidemic in region**										
**Days** ***N***	**Threshold** ***ϖ***	**1**	**2**	**3**	**4**	**5**	**6**	**7**	**8**	**9**	**10**	**11**
1	.2	8/22	8/22	8/22	8/22	8/22	8/22	8/22	8/22	8/22	8/22	8/22
1	.5	8/23	8/23	8/22	8/22	8/22	8/22	8/22	8/23	8/22	8/23	8/23
1	.8	8/23	8/23	8/22	8/23	8/22	8/23	8/23	8/23	8/22	8/23	8/23
2	.2	8/22	8/22	8/22	8/22	8/22	8/22	8/22	8/22	8/22	8/22	8/22
2	.5	8/23	8/23	8/22	8/22	8/22	8/22	8/22	8/23	8/22	8/23	8/23
2	.8	8/23	8/23	8/22	8/23	8/22	8/23	8/23	8/23	8/22	8/23	8/23
3	.2	8/22	8/22	8/22	8/22	8/22	8/22	8/22	8/22	8/22	8/22	8/22
3	.5	8/23	8/23	8/22	8/22	8/22	8/22	8/22	8/23	8/22	8/23	8/23
3	.8	8/23	8/23	8/22	8/23	8/22	8/23	8/23	8/23	8/22	8/23	8/23

The epidemic is declared when *P*(*δ*(*t*) = 1*Y*_1:*t*_) exceeds the threshold *ϖ* for *N* consecutive days.

We stress that these rules are simply an illustration of how the quantitative outputs of our model applied to a real data setting, and how they illuminate the false positive ‐ false negative tradeoffs that public health agencies make. We did not attempt to address the complexities in real life, which include loss functions, limited resources, other sources of information, how agencies interpret and react to uncertainty, and data quality issues, amongothers.

## Conclusions

In this paper, we used a Bayesian methodology that adapts the existing Gaussian Markov random fields class of models to accommodate spatio‐temporal surveillance data. By applying this methodology to real data, we gained insights into both the methodology and the real world problems. Features of the model include timely detection of outbreaks, robust inference to model misspecification, reasonable prediction performance, and analytical results and visualization to assist public health authorities in risk assessment.

Controlling false positives is a critical issue in a real surveillance setting, and a proper decision rule is the key. One can avoid this issue in the Bayesian formulation by considering the posterior joint distribution to control the overall false alarms (cf. Scott and Berger [20]). We control false positives by introducing the variables *δ*_*i*_(*t*), which indicate an outbreak. From an operational aspect, we illustrate a decision rule under which an alarm is sounded only when the posterior probability is greater than a certain threshold *ϖ* for *N* consecutive days. A sensitivity analysis based on the decision rule for the PHESS data set was carried out and highlighted to show the impact of the settings chosen (false positive vs. waiting too long to report an outbreak). Additional simulations on this topic are planned in a parallel study. Based on the real data applications, we demonstrated that the model is capable of capturing outbreaks rapidly, while still limiting falsepositives.

Using our methodology, we analyze real surveillance data consisting of 2008–2010 Indiana respiratory syndrome counts from the PHESS data set. A three‐part case study was presented in this article that has unique and interesting outbreak patterns. Finally, the advantages of our methodology for addressing the complicated issues of real world surveillance data applications are three‐fold. We can easily incorporate additional covariate information and spatio‐temporal dynamics in the data. Second, we furnish a unified framework to provide uncertainties associated with each parameter. Third, we are able to handle multiplicity issues by using a Bayesian approach. The urgent need to quickly and effectively monitor the health of the public makes our methodology a potentially plausible and useful surveillance approach for health professionals.
